# Adaptive vaccination strategies to mitigate pandemic influenza: Mexico as a case study

**DOI:** 10.1371/currents.RRN1004

**Published:** 2009-08-18

**Authors:** Gerardo Chowell, Cecile Viboud, Xiaohong Wang, Stefano Bertozzi, Mark Miller

**Affiliations:** ^*^Arizona State University; ^†^Fogarty International Center, National Institutes of Health, Bethesda, MD, USA; ^§^National Institute of Public Health, Cuernavaca, Mexico; University of California, Berkeley, USA and ^¶^National Institutes of Health

## Abstract

In this modeling work, we explore the effectiveness of various age-targeted vaccination strategies to mitigate hospitalization and mortality from pandemic influenza, assuming limited vaccine supplies. We propose a novel adaptive vaccination strategy in which vaccination is initiated during the outbreak and priority groups are identified based on real-time epidemiological data monitoring age-specific risk of hospitalization and death. We apply this strategy to detailed epidemiological and demographic data collected during the recent swine A/H1N1 outbreak in Mexico. We show that the adaptive strategy targeting age groups 6-59 years is the most effective in reducing hospitalizations and deaths, as compared with a more traditional strategy used in the control of seasonal influenza and targeting children under 5 and seniors over 65. Results are robust to a number of assumptions and could provide guidance to many nations facing a recrudescence of A/H1N1v pandemic activity in the fall and likely vaccine shortages.

## Past research on influenza vaccination strategies

Given the variety of possible pandemic scenarios and issues with rapid production of influenza vaccines [1], information on age patterns of incidence and mortality during the early phase of a pandemic could help prioritize allocation of limited vaccine resources, and optimize disease burden reductions. Control strategies for influenza pandemics have been explored by simulations in several countries or regions, including Southeast Asia [2] [3], US [4] [5] [6] [7], UK [7], and Netherlands[6] [7] [8]. None of these simulations featured adaptive age-targeted vaccination strategies that integrate epidemiological data collected in real time during the first weeks of the outbreak. The recent emergence of a novel swine-origin influenza A/H1N1 virus (S-OIV) in spring 2009 in Mexico [9] and rapid global spread [10] provides an opportunity for real-time modeling of an on-going pandemic. Such modeling work may help define priority age groups for vaccine allocation in the coming months, as the next wave of the A/H1N1 pandemic hits the Northern Hemisphere countries.

****

## S-OIV H1N1 pandemic scenario assuming limited vaccine supplies

Here, we explore age-targeted vaccination strategies against pandemic influenza in Mexico based on a mathematical transmission model rooted in epidemiological data from past influenza pandemics and the novel A/H1N1 outbreak.  In the context of limited vaccine supplies, we propose a novel ‘adaptive’ vaccination strategy that allocates vaccines based on age-specific rates of hospitalization or mortality monitored in the early stages of the pandemic. We show that the ‘adaptive’ strategy provides robust benefits and is consistently superior to the ‘seasonal’ vaccination strategy, which relies on the traditional priority groups defined for seasonal influenza, ie, young children (0-5 y) and persons over 60 years of age. 

## Description of modeling approach

In this simulation approach, we assumed limited vaccine supplies, as well as age variation in vaccine efficacy, risk of illness, hospitalization, and death from pandemic influenza using a transmission model that includes 6 age groups (0-5 y, 6-12 y, 13-19 y, 20-39 y, 40-59 y, >=60 y). To compare the effectiveness of various vaccination strategies, we used an age-structured influenza transmission model (Figure 1) that integrates risk of illness, hospitalization, and death. Population size and age structure was based on the Mexican census for year 2000 [11]. Transmission between age groups was modeled based on a study describing self-reported age-specific contact rates for the spread of respiratory infections in the Netherlands [12]. Although information on contact rates is limited [13], these self-reported contact rates have been shown to provide better approximations to attack rates of the 1957 influenza pandemic than other mixing assumptions [12]. The contact rate matrix was highly assortative with higher mixing rates within each age group than between age groups and rates followed a similar pattern to those described for several European countries [14]. 

**Figure:**
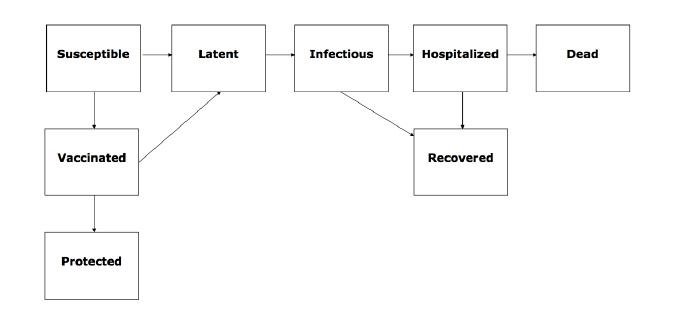
**Figure 1: **Flow chart of the state progression of individuals among different epidemiological classes assumed by our age-structured influenza transmission model.  

The basic reproduction number R_0_, the number of secondary cases generated by a primary infectious case [15] [16], quantifies the transmissibility of a pathogen in naïve populations, while the reproduction number, R, estimates a similar quantity in partially-naïve populations.  These estimates can help determine the intensity of interventions necessary for epidemic control. If the average number of secondary infections is reduced then transmission slows down (transmission is interrupted if R<1), so that there is less pressure on health care systems and potentially increased time to prepare additional vaccines. We derive an expression of the reproduction number from our mathematical model using standard methods [16] and calibrate our model against published estimates of the basic reproduction number for the S-OIV outbreak in Mexico.

In these simulations, vaccination strategies are implemented after the onset of an influenza pandemic outbreak. We considered 2 strategies, depending on the age targets for vaccination: 1. an ‘adaptive’ vaccination strategy, where vaccine is allocated on the basis of age-specific rates of hospitalization and mortality reported in real-time during the early pandemic phase.  More specifically, vaccine doses are allocated to each age group proportionally to their corresponding hospitalization or mortality rates. For example, if the hospitalization rate was 10-fold higher in young adults than in young children, then young adults would experiecne a 10-fold higher vaccination rate than young children. Benefits are potentially optimized if early reports of age-specific hospitalization and death are indicative of those at risk throughout all phases of a pandemic. 2. For reference purposes, we also considered a ‘seasonal’ influenza vaccination strategy targeting the same age groups as for seasonal vaccination, ie, young children (0-5 y) and seniors (>=60 y). With the seasonal vaccination strategy, vaccination rate is set to be equal in those extreme age groups, while it is set at 0% in intermediate age groups. 

To simulate realistic epidemiological patterns associated with the S-OIV influenza pandemic, we used age-specific data reported to the Mexican National Epidemiological Surveillance System during March-April 2009 [17]. Mean baseline values for the latency and infectious period were assumed to be 1.5 days (range 1-4 days) in agreement with previous studies [2] [18]. Age-specific hospitalization and case fatality rates for hospitalized cases (age groups 0-5 y, 6-59 y, >=60 y) were estimated from cumulative morbidity and mortality data reported on two epidemiologically-relevant dates of the outbreak: (i) April 17, when the Mexican Ministry of Health requested that medical institutions intensify case notification, 25 days into the outbreak (March 24 -April 17, 2009)  (ii) on Apr 29 when selective reporting of pneumonia requiring hospitalization ceased (Figure 2), which happened 37 days into the outbreak [9]. We varied the mean reproduction number within its estimated range of 1.4-1.8 [10].

We assumed that timing of vaccine delivery followed an exponential distribution with an average of 5 days after the vaccination campaign was initiated. Vaccine efficacy was assumed to be 77.5% (range between 75% and 80%) for individuals < 60 y and 35% (range between 17% and 53%) for seniors over 60 years, based on reviews of influenza vaccine immunogenicity [19] [20] [21]. Vaccinated individuals were assumed to develop protection 10 or 30 days after immunization on average, depending on the requirements of one or two doses of a novel vaccine. Because vaccine resources will likely be scarce during the early phase of the next pandemic wave, we assumed a relatively low vaccination coverage with the full course of 1-2 doses (5% - 20%). Our upper bound for vaccination coverage was consistent with the immunization of about one fifth of the US population in 1976 against a swine influenza virus [22]. We assumed that the same vaccination strategy would be applied throughout the outbreak or until vaccines resources were depleted.

## Results

### Baseline Scenario 

First, we explored a baseline pandemic scenario reminiscent of the recent Mexican experience with novel S-OIV in the spring of 2009, in the absence of any intervention. Assuming R_0_ = 1.6, the peak was reached at about day 120 after the pandemic onset with a total outbreak duration of about 5 months. Hospitalization rates varied from about 10% for the 13-19 year-olds to 17% for the >=60 year-olds. Mortality rates were estimated at about 2.3-, 1.9- and 0.7-percent for year age groups 20-39, 13-19, and >=60 y, respectively. 

****

### Benefits of the adpative vaccination strategy 

To define priority groups for the adaptive strategy, we relied on early estimates of age-specific rates of hospitalization and death reported on days 25 and 37 of the Spring 2009 outbreak in Mexico (Fig. 2). Given the age patterns of hospitalizations and deaths in the S-OIV Mexican outbreak, middle age group  6-59 yo should be prioritized for vaccination. Specifically, these age groups should receive 70% of the vaccine stockpile if the goal is to hospitalizations, or 93% of the stockpile if the goal is to minimize deaths.  

**Figure:**
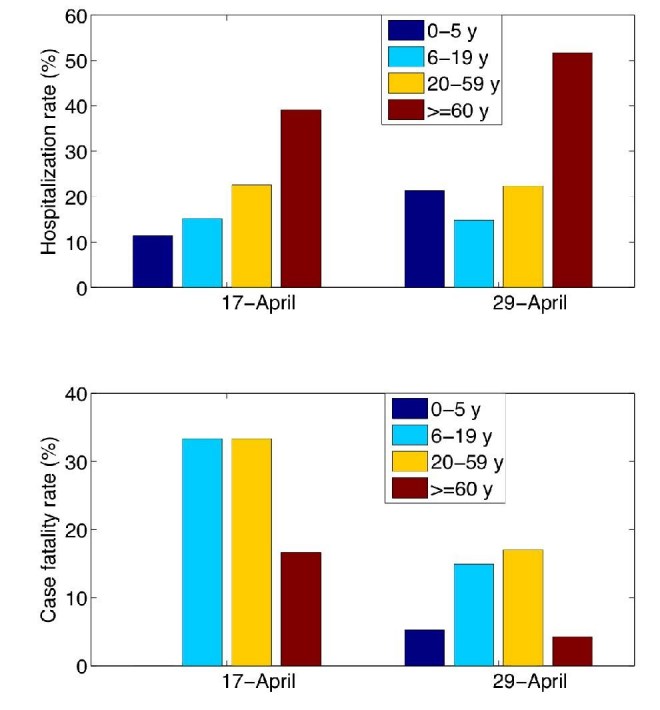
**Figure 2:**  Age-specific rates of hospitalization and death among clinical cases during the S-OIV outbreak in Mexico. Rates are estimated from cumulative morbidity and mortality data provided by the National Surveillance System on April 17 (epidemiological alert) and April 29, 2009 (end of selective reporting of severe pneumonia cases). Hospitalization rate is estimated as the proportion of hospitalizations among pneumonia cases and mortality rate is estimated as the proportion of deaths among hospitalized pneumonia cases.** ** 

Assuming R_0_=1.6 (Table 1), the adaptive strategy yielded reductions of 37% and 42% in the overall number of hospitalizations and deaths, respectively, if vaccination started on day 25 of the outbreak and reached 20% of the population. The benefits of the adaptive strategy were slightly lower if vaccination started on day 37 of the outbreak and reached 20% of the population. 

**Figure:**
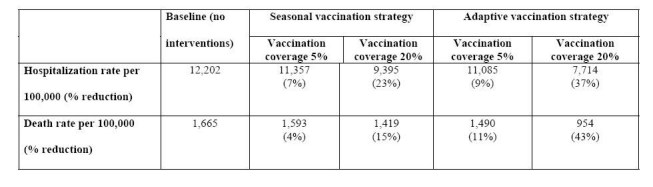

Table 1.Comparison of predicted rates of hospitalizations and deaths associated with the 2009 S-OIV influenza pandemic under various vaccination scenarios. We considered a baseline scenario where no vaccine is used; a seasonal vaccination strategy where priority groups for vaccination are young children under 5yrs and seniors > 60yrs, as for seasonal influenza; and an adaptive vaccination strategy where vaccine is allocated according to age-specific rates of mortality and hospitalization estimated in real-time. Hospitalization and death rates are given per 100,000 people; numbers in parentheses indicate percentage burden reduction as compared with the baseline non-intervention scenario. We assumed that R0=1.6 [10] , vaccine resources were very limited so that coverage remained low at 5 or 20% of the overall population, and vaccination was initiated 25 days after the epidemic onset.

****

### Comparison with the traditional "seasonal" vaccination strategy

The adaptive vaccination strategy always outperformed the seasonal strategy, and provided up to 35% additional reduction in the number of influenza-related deaths and 22% reduction in hospitalization, for transmissibility levels of 1.4-1.8 and vaccination coverage ranging between 5 and 20% (Figure 3).  Overall, the additional benefits of the adaptive strategy, relative to the seasonal strategy, were larger for earlier vaccination campaigns, higher vaccination rates, and lower R0 values.  

**Figure:**
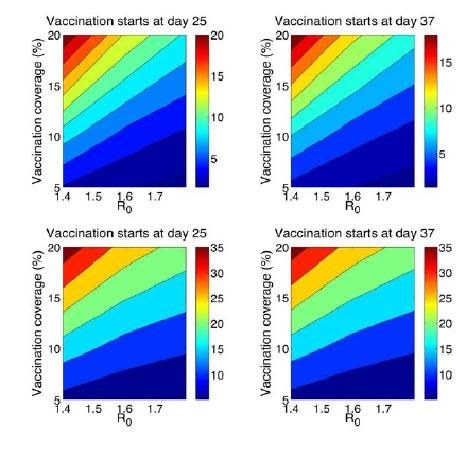
** Figure 3:   **Comparison of adaptive and seasonal vaccination strategies to mitigate deaths and hospitalizations associated with novel S-OIV pandemic influenza. The adaptive vaccination strategy prioritizes age groups proportionally to age-specific rates of hospitalization and death estimated in real-time, while the seasonal vaccination strategy targets young children under 5 and seniors over 60. Plots illustrate the additional reduction (%, see color bar) in hospitalizations (top) and deaths (bottom) averted by the adaptive vaccination allocation strategy, compared to the seasonal influenza strategy, as a function of R (using plausible ranges, R=1.4-1.8) and vaccination coverage (5-20%). In the left panels, vaccination is initiated 25 days into the outbreak, on April-17-2009, when the epidemiologic alert was issued.  In the right panels, vaccination is initiated 37 days into the outbreak, on Apr-29-2009, when selective reporting of severe pneumonia cases ceased. 

## Discussion

In this study, we propose a novel adaptive vaccination strategy to mitigate deaths and hospitalizations associated with pandemic influenza when vaccine resources are scarce, using real-time epidemiological data collected early in the outbreak. Our simulations calibrated against the transmission dynamics and epidemiology of the Spring 2009 S-OIV outbreak in Mexico showed that the adaptive strategy was the most effective and substantially outperformed the seasonal influenza allocation strategy for a range of parameter values. In particular, the adaptive strategy would provide additional reductions of up to 22% and 35% in hospitalizations and deaths compared to the ‘seasonal’ vaccination strategy targeting traditional high-risk groups, if vaccination was initiated 25 days into the outbreak and reached 20% of the population. Based on the epidemiology of the S-OIV pandemic so far, the population between 20 and 59 years would be preferentially targeted with the adaptive strategy, in contrast to seniors and young children who are traditionally prioritized for control of seasonal influenza.

An adaptive vaccination strategy requires rapid ascertainment of cases, hospitalizations and/or deaths, to help identify high risk age groups for prioritization of vaccine and other pharmaceutical interventions, including antivirals and antibiotics [23] [24]. In the 2009 S-OIV influenza outbreak, the optimal strategy could be identified with confidence as early as day 25 of the Mexican outbreak, given knowledge on the age pattern of severe cases and local availability of real-time data.  Had a vaccine been available in quantities sufficient to cover 20% of the population, this would have given enough time to initiate a vaccination campaign and avert an additional 22% of hospitalizations and 35% of deaths compared to a seasonal vaccination strategy if vaccination started 25 days after the epidemic onset. Alternatively, if local epidemiological data are not available in real time, data from other countries experiencing earlier or simultaneous outbreaks could be used to calibrate the adaptive strategy. Such a strategy might be particularly useful in the case of returning outbreaks of S-OIV in the fall, in Mexico and elsewhere.

Virological subtyping of a novel pandemic virus can provide an early clue to target vaccination efforts.  Each of the previous pandemics had unique age-mortality patterns [1] [25] that could be explained by previous exposure during childhood of a subset of the population to the novel circulating viral sub-type [26] [27]. While the elderly are normally at most risk for severe outcomes during seasonal influenza, warranting the targeting of vaccination for direct protection to that group, they may have residual protection during pandemics. By contrast, younger groups generally respond better to vaccine [19] [20] [21] and provide a greater reduction of transmission. Given residual protection in seniors in early pandemic waves, younger age groups become a clear priority group for pandemic vaccine allocation. In the current 2009 pandemic, inidviduals born between 1919 and around 1957 would have been first exposed to H1N1 during their childhood and may enjoy protection against S-OIV infection and death, as observed in the early wave of S-OIV in Mexico [9].

There are many limitations to policy models with respect to choice of parameter estimates and the incorporation of bio-medical, environmental, operational, political, economic features.  No single model can claim to incorporate all assumptions and features given the limited data and uncertainty associated with influenza pandemics.   Our model illustrates a prioritization scheme based on age-groups but does not further discriminate other sub-groups such as those persons with other medical conditions including pregnancy. Models do not necessarily provide answers but help articulate the questions, assumptions and numerous uncertainties in rapidly evolving circumstances and should be viewed as tools to formulate rational policy based on the best available evidence.  Pandemics evolve rapidly relative to capabilities to enact policies; therefore, pre-formulated adaptive strategies can readily take into account new data.   Knowledge of the specific sub-type circulating and real-time information on age-specific rates of severe outcomes are crucial to help policy makers infer who may be at most risk, and tailor intervention strategies accordingly.  These adaptive pandemic strategies could be readily adopted by other countries.

##  Acknowledgments

We thank Raymond Hutubessy, WHO, for continued support, and Bryan Grenfell, Princeton University and Fogarty International Center, National Institutes of Health, for helpful suggestions.

## Funding information

The study was funded by the Quantitative Immunization and Vaccine-Related Research (QUIVER) program of the WHO and the intra-mural program of the Fogarty International Center,  US National Institutes of Health. 

## Competing interests 

The authors have declared that no competing interests exist.
